# Expression of microRNAs related to apoptosis in the aqueous humor and lens capsule of patients with glaucoma

**DOI:** 10.3389/fmed.2024.1288854

**Published:** 2024-02-14

**Authors:** Hyo Seon Yu, Eun Hee Hong, Ji Hye Kang, Yong Woo Lee, Won June Lee, Min Ho Kang, Heeyoon Cho, Yong Un Shin, Mincheol Seong

**Affiliations:** ^1^Department of Ophthalmology, Hanyang University College of Medicine, Seoul, Republic of Korea; ^2^Department of Ophthalmology, Hanyang University Guri Hospital, Guri, Gyeonggi-do, Republic of Korea; ^3^Hanyang Institute of Bioscience and Biotechnology, Hanyang University, Seoul, Republic of Korea; ^4^Graduate School of Biomedical Science & Engineering, Hanyang University, Seoul, Republic of Korea; ^5^Department of Ophthalmology, Kangwon National University Graduate School of Medicine, Kangwon National University Hospital, Chuncheon, Republic of Korea; ^6^Department of Ophthalmology, Hanyang University Hospital, Hanyang University College of Medicine, Seoul, Republic of Korea; ^7^NOON Eye Clinic, Guri, Gyeonggi-do, Republic of Korea

**Keywords:** microRNA, glaucoma, aqueous humor, lens capsule, biomarker

## Abstract

**Background:**

The aim of this study is to investigate the expression profiles of microRNAs (miRNAs) related to apoptosis in the aqueous humor (AH) and lens capsule (LC) of patients with glaucoma.

**Methods:**

AH and LC samples were collected from patients with open-angle glaucoma and control participants who were scheduled for cataract surgery. A miRNA PCR array comprising 84 miRNAs was used to analyze the AH (glaucoma, *n* = 3; control, *n* = 3) and LC samples (glaucoma, *n* = 3; control, *n* = 4). Additionally, the AH and LC samples (glaucoma, *n* = 3; control, *n* = 4) were subjected to quantitative real-time PCR to validate the differentially expressed miRNAs determined using the PCR array. Bioinformatics analysis was performed to identify the interactions between miRNAs and diseases. Additionally, the differential expression of these miRNAs and the target gene was validated through *in vitro* experiments using a retinal ganglion cell (RGC) model.

**Results:**

Expression levels of 19 and 3 miRNAs were significantly upregulated in the AH and LC samples of the glaucoma group, respectively (*p* < 0.05). Of these, the expression levels of hsa-miR-193a-5p and hsa-miR-222-3p showed significant differences in both AH and LC samples. Bioinformatics analysis showed experimentally validated 8 miRNA:gene pairs. Among them, *PTEN* was selected to analyze the expression level in AH and LC from separate cohort (glaucoma, n = 5; control, *n* = 4). The result showed downregulation of *PTEN* concurrent with upregulation of the two miRNAs in LC samples of glaucoma group. *In vitro* experiments validated that the expression levels of hsa-miR-193a-5p and hsa-miR-222-3p were significantly upregulated, and that of *PTEN* was significantly downregulated in the H_2_O_2_-treated RGC, while the level of *PTEN* was recovered through co-treatment with miR-193a inhibitor or miR-222 inhibitor.

**Conclusion:**

This is the first study to investigate the differential expression of apoptosis-related miRNAs in the AH and LC of patients with glaucoma. Hsa-miR-193a-5p and hsa-miR-222-3p, which were upregulated in both AH and LC, may be considered potential biomarkers for glaucoma.

## 1 Introduction

Glaucoma, a neurodegenerative disease, is characterized by retinal ganglion cell (RGC) death and optic nerve damage (1). The representative risk factors for glaucoma include aging, high intraocular pressure (IOP), and a family history of glaucoma (2). In particular, high IOP is a major risk factor that is regulated by the circulation of aqueous humor (AH). The damage or dysfunction of trabecular meshwork (TM) tissue, a passage through which AH flows, dysregulates the outflow of AH and consequently increases the IOP (3). Previous studies have reported TM alterations in eyes with glaucoma, which involve decreased cellularity (4) and the accumulation of apoptotic cells in TM tissue (5, 6). Functional and microstructural changes in TM tissue and TM cell apoptosis are among the significant pathological changes observed in open angle glaucoma (OAG) (7).

MicroRNAs (miRNAs), which are single-stranded non-coding RNAs with a length of approximately 22 nucleotides, regulate biological signals in various diseases by inhibiting the transcription of target genes (8). Growing evidence supports the role of miRNAs in the pathogenesis of neurodegenerative diseases, such as glaucoma, Alzheimer’s disease, and Parkinson’s disease, by regulating genes related to extracellular matrix (ECM)/cell proliferation, the immune system, and regulation of apoptosis (9, 10). MiRNAs play a crucial role in regulating apoptosis in various diseases (11), and their role in TM cell apoptosis in OAG has been actively investigated (12). Previous studies have explored differentially expressed miRNAs in glaucoma using peripheral blood mononuclear cell, plasma, and AH samples (13, 14). However, the expression profiles of miRNAs related to apoptosis in the lens capsule (LC) together with AH have not been analyzed in patients with OAG, except for those with lens-related glaucoma.

AH is continuously produced by the ciliary body and secreted out of the eye after directly coming in contact with the lens, iris, and surface of the corneal endothelium (15). As a body fluid secreted by many cells, AH can inform on conditions of the eye structures, such as lens epithelial cells and corneal endothelium, and the metabolites of the retina (16). The anterior LC lies in the monolayer subcapsular lens epithelium, which is the most important metabolic part of the lens (17). AH is in direct contact with the anterior LC and TM. Therefore, apoptotic factors in the AH can also affect or indicate the status of LC and TM. Moreover, LC may be an indirect indicator of the effect of AH cytokine/metabolites on the TM tissue. In contrast to AH which contains only extracellular biomaterials, the LC samples allow direct detection of biomaterials (such as microRNAs) from lens epithelial cells, providing useful insights into pathological mechanisms. Therefore, it may be helpful to evaluate the status of LC and AH simultaneously in patients with glaucoma.

In this study, we investigated the expression profiles of miRNAs associated with apoptosis in AH and LC samples obtained from patients with glaucoma. Furthermore, the potential mechanisms of differentially expressed miRNAs in glaucoma were examined based on their related proteins and signaling pathways.

## 2 Materials and methods

This prospective cross-sectional study included control participants and patients with glaucoma who visited the Department of Ophthalmology of Hanyang University Guri Hospital, Gyeonggi-do, South Korea between November 2019 and May 2021. The institutional review board (IRB) of Hanyang University Guri Hospital reviewed and approved the study protocol (IRB file no. 2019–05-022) and the protocol of this study adhered to the tenets of the Declaration of Helsinki. All study participants provided written informed consent to participate in this study.

### 2.1 Participants

This study recruited 29 participants who were scheduled for cataract surgery: 14 patients with open-angle glaucoma and 15 control participants. Glaucoma was diagnosed by a glaucoma specialist based on the clinical examination of the glaucomatous optic nerve head associated with typical and reproducible visual field defects. Glaucomatous visual field defects on the standard automated perimetry were defined based on a glaucoma hemifield test result outside the normal limits and the presence of at least three contiguous test points within the same hemifield on the pattern deviation plot at *p* < 1% (at least one point at *p* < 0.5%) in at least two consecutive tests with reliability indices better than 15%. The inclusion criteria for patients with glaucoma were as follows: no history of ocular diseases other than glaucoma and cataract, no prior intraocular surgery, and no systemic diseases other than hypertension. Patients diagnosed with lens-related glaucoma using the slit lamp examination were excluded. The inclusion criteria for control participants were as follows: no prior intraocular surgery, no history of ocular diseases other than cataract, no use of any topical ocular medications, except the use of preoperative topical mydriatics and antibiotics, and no systemic diseases other than hypertension. The exclusion criteria for study subjects in both groups were as follows: received medication other than antihypertensive medication at any time during the study, a history of stroke or myocardial infarction, collection of insufficient amount of AH sample for analysis, or AH sample was judged as inappropriate for analysis.

All participants underwent standard ophthalmologic examinations, including IOP measurement, best-corrected visual acuity, slit lamp biomicroscopy, optical coherence tomography (swept source OCT, Topcon DRI OCT-1 Atlantis; Topcon, Inc., Tokyo, Japan), and Optos ultra-wide fundus photography (Optos, Dunfermline, Scotland). Patients with glaucoma additionally underwent gonioscopy, ultrasonic central corneal thickness measurements, IOP evaluation with Goldmann applanation tonometry, stereoscopic optic nerve head examination, and visual field examination (30–2 strategy on Humphrey Perimeter).

### 2.2 Sample collection

AH samples were collected by the same operator (MS) at the start of the cataract surgery. Briefly, one or two drops of 0.5% proparacaine hydrochloride (Alcaine, Alcon, Ft. Worth, TX, United States) and 5% povidone-iodine were instilled after placing a sterile eyelid speculum. At the beginning of cataract surgery, anterior chamber paracentesis was performed under the surgical microscope using a 30-gauge needle mounted on a 1 mL tuberculin syringe to collect approximately 50–200 μL of AH. LC samples were obtained from the central anterior capsules of the lens (5–6 mm in diameter) via capsulorhexis during cataract surgery. Intact continuous curvilinear capsulorhexis was performed, avoiding vascular contact or damage to the iris and other intraocular structures. All AH and LC samples were transferred to tubes and immediately frozen at −80°C until further processing.

### 2.3 miRNA extraction and complementary DNA (cDNA) synthesis

#### 2.3.1 Preparation of AH sample for miRNA polymerase chain reaction (PCR) array

miRNAs from AH samples were extracted using the miRNeasy serum/plasma kit (Qiagen, Hilden, Germany). For normalization, miRNeasy serum/plasma spike-in control (1.6 × 10^8^ copies/μL; Qiagen) was added to all samples, following the manufacturer’s instructions. RNA (45 ng) from AH samples was mixed with the miScript II RT kit (Qiagen) for cDNA synthesis according to manufacturer’s instructions. The mixtures were incubated at 37°C for 60 min and 95°C for 5 min. The cDNA was immediately diluted in 20 μL of RNase-free water and stored at −20°C until use.

To perform the miRNA PCR array analysis, cDNA prepared from the AH samples was pre-amplified in a reaction mixture comprising Hot-Start Taq DNA polymerase, miScript PreAMP universal primers, miScript PreAMP buffer, miScript PreAMP primer mix, and RNase-free water in the miScript PreAMP PCR kit (Qiagen). The mixtures were incubated at 95°C for 15 min, followed by 21 cycles of 94°C for 30 s and 60°C for 3 min. The pre-amplified cDNA was diluted in 225 μL RNase-free water.

Real-time PCR was performed to confirm the quality of the pre-amplified cDNA. A 10× *Caenorhabditis elegans* miR-39 miScript primer, a 10× miRTC miScript primer, and a 10× miR-16 miScript primer were mixed with 2× QuantiTect SYBR Green PCR master mix, 10× miScript universal primer, and RNase-free water from the miScript PreAMP PCR kit and miScript SYBR Green PCR kit (Qiagen). The threshold cycle (Ct) values of the pre-amplification controls were determined.

#### 2.3.2 Preparation of LC sample for miRNA PCR array

miRNAs from the LC samples were isolated using the miRNeasy micro kit (Qiagen). LC RNA samples (150 ng) were mixed with the miScript II RT kit (Qiagen) for cDNA synthesis. The cDNA was immediately diluted to perform miScript microarray analysis and stored at −20°C until use.

### 2.4 miRNA PCR array

A PCR array panel was used to identify the candidate miRNAs. Among several miRNA PCR array panels, a panel comprising miRNAs related to human apoptosis was selected, as apoptosis is one of the main alterations in TM tissues in eyes with glaucoma. miRNA PCR array was performed with an miScript miRNA PCR Array Human apoptosis kit (96-well format, Qiagen, #MIHS-114ZC) with 43 cycles, according to the manufacturer’s specifications. For the AH samples, miRNA PCR array results were calibrated with cel-miR-39 and miRTC (miRNA reverse transcription control) and simultaneously normalized with RNU6-6P. Meanwhile, for the LC samples, the miRNA PCR array results were normalized with SNORD95 (Small Nucleolar RNA, C/D Box 95). A list of 84 genes from the human apoptosis PCR array panel, excluding 12 controls, is provided in Supplementary Table S1. miRNA expression was analyzed using the Qiagen Data Analysis Center.^1^ The results are presented in the form of clustergrams and heatmaps, which are generated using the online Qiagen Data Analysis Center.^2^

### 2.5 Quantitative real-time PCR (qRT-PCR)

Total RNA was extracted from the AH and LC samples using the same protocol as described above. cDNA was synthesized using the miRCURY LNA RT kit (Qiagen) according to manufacturer’s instructions. To validate the differential expression of hsa-miR-193a-5p and hsa-miR-222-3p determined using the PCR array, commercially available PCR primer mixes (Qiagen) were used with the miRCURY LNA miRNA PCR assay (Qiagen), following the manufacturer’s instructions. UniSp6 expression was used as an internal control. Fluorescence data were collected, and the relative expression of miRNAs was calculated using delta C_T_.

### 2.6 Reverse transcription-PCR (RT-PCR)

cDNA was synthesized using amfiRivert cDNA Synthesis Platinum Master Mix (GenDEPOT) according to the manufacturer’s protocol. The RT-PCR was performed with Taq DNA polymerase (GenDEPOT) on a thermal controller (Applied Biosystems). The PCR reaction was initiated with a 2 min incubation at 94°C, followed by 40 cycles of denaturation at 94°C for 15 s, annealing at 55°C for 30 s, and extension at 72°C for 30 s, and terminated after a 10 min extension at 72°C. Each PCR product (5 μL) was electrophoresed in a 1.5% agarose gel, and bands were visualized using the SafePinky DNA Gel Staining Solution (GenDEPOT). The densities of the DNA bands were analyzed using an image analyzer (ImageQuant LAS 4000).

### 2.7 Bioinformatics

#### 2.7.1 Prediction the experimentally validated miRNA target genes

Target annotation analysis was performed using R (version 3.6.3),^3^ and appropriate packages according to the corresponding reference manuals. Identification of miRNA-gene regulatory interactions was performed in silico between selected miRNAs and glaucoma-associated genes harvested from DisGeNET 7.0 database^4^ (18), (using multiMiR 1.10.0 package,^5^ database version: 2.3.0, updated on 2020–04-15) (19, 20). The analysis included the identification of both experimentally validated (miRecords, miRTarBase, and TarBase databases) and predicted (DIANA-microT-CDS, ElMMo, MicroCosm, miRanda, miRDB, PicTar, PITA, and TargetScan databases) miRNA-gene interactions. Among the identified miRNA-gene regulatory interactions, those validated based on experiments known as strong evidence, including western blotting, qRT-PCR, and reporter assays, were included in target gene selection for expression level analysis using AH and LC samples and gene ontology enrichment analysis to identify significant pathways.

#### 2.7.2 Gene ontology enrichment analysis

The interactions of validated target genes were analyzed using ClueGO in Cytoscape. The enrichment analysis revealed a list of statistically significant genes and the connection between biological processes. Pathway networks and gene ontology terms were identified using Kyoto Encyclopedia of Genes and Genomes (KEGG) (21), biological process, and Reactome database of protein complex in ClueGO. All images represent statistically significant pathways (*p* < 0.05).

#### 2.7.3 Bioinformatics analysis with Laverne

The relationship between miRNA genes and glaucoma was analyzed using the Laverne Bioinformatics Tool from Novus Biologicals.^6^ The lines of the bioinformatic images are based on the supporting evidence.

### 2.8 *In vitro* experiment

To validate the findings from samples of patients with glaucoma, *in vitro* experiments using a glaucomatous cell model (RGC) were conducted.

#### 2.8.1 Primary RGC isolation

A total of 28 pregnant Sprague Dawley rats were purchased form Orientbio (Gyeonggi-do, South Korea), and 280 2-days old rats were used for RGC isolation. Newborn rats were euthanized by decapitation, conducted by an expert to minimize the number and pain of animals used in the experiment. Each experiment was conducted in duplicate and repeated three or four times from different cell harvests. Retinal tissues of 2-days old rats were incubated in Hank’s balanced salt solution (Gibco) containing 5 mg/mL of papain (Sigma), 0.24 mg/mL of L-cysteine (Sigma), 0.5 mmoL/L of EDTA (Sigma), and 10 U/mL of DNase І (Worthington) for 30 min. Dissociated retinal cells were collected as a suspension. The retinal cell suspension was incubated with rabbit anti-rat macrophage antibody (1:50 dilution; Fitzgerald) for 5 min. The suspension was treated in a 100-mm Petri dish coated with goat anti-rabbit antibody (1:200 dilution; Jackson laboratory) for 30 min. Non-adherent cells were incubated with anti-Thy1 microbeads (1:10, Miltenyi Biotec) for 30 min at 4°C, and the magnetic-labeled RGCs were collected using a magnetic separating unit. All procedures were performed under the approve of Institutional Animal Care and Use Committee (IACUC) of Hanyang university.

#### 2.8.2 RGC culture and hydrogen peroxide (H_2_O_2_) treatment

The cells were cultured in neurobasal media (Gibco) containing 1% penicillin/streptomycin (Gibco), B-27^™^ Supplement (50X, Gibco), 4.0 mg/L of Forskolin (Sigma), 80 mg/L of brain-derived neurotrophic factor, and 80 mg/L of ciliary neurotrophic factor. Cells were seeded on a 6-well plate precoated with poly-L-ornithine and laminin. The seeding density was approximately 1 × 10^6^ cells per well. The cultures were incubated at 37°C in humidified 5% CO_2_ and 95% air. To measure changes in the expression levels of the miRNAs and *PTEN*, the RGCs (1 × 10^6^/well) were exposed to 100 μM H_2_O_2_ for 6 h in a 6-well plate.

#### 2.8.3 miRNA regulation

Transfection of inhibitors of the miRNAs were performed a day before H_2_O_2_ treatment. 20 pmol rno-miR-193a inhibitor (Invitrogen) and 20 pmol rno-miR-222 inhibitor (Invitrogen) were used for transfection. Inhibitors were incubated for 20 min at room temperature before transfection. The inhibitors were transfected into the RGC using lipofectamine RNAiMAX Transfection Reagent (Invitrogen) for 24 h, and then transfected-RGCs were treated with H_2_O_2_. All cells were collected for RT-PCR analysis.

### 2.9 Statistical analysis

All data are presented as mean ± standard deviation from at least three independent experiments. The means of different groups were compared using Student’s t-test. Differences were considered significant at *p* < 0.05. Correlation analysis between AH and LC samples was performed using Spearman correlation analysis.

## 3 Results

Among 14 patients with glaucoma and 15 control participants, the AH samples from 3 patients with glaucoma and 3 control participants and the LC samples from 3 patients with glaucoma and 4 control participants were used for miRNA PCR array analysis. Additionally, both AH and LC samples from 3 patients with glaucoma and 4 control participants were used for qRT-PCR analysis. AH and LC samples from the remaining 5 patients with glaucoma and 4 control participants were used for RT-PCR of *PTEN* and qRT-PCR of miRNAs. Table 1 shows the baseline clinical characteristics of enrolled participants.

**Table 1:** Characteristics and types of experiment and sample of study participants.

	Sex	Age (years)	Diagnosis	Laterality	Underlying disease	miRNA PCR array_AH	miRNA PCR array_LC	miRNA qRT-PCR*	RT-PCR, miRNA qRT-PCR^†^
Control									
1	Female	73	Cataract	OS	None	√			
2	Female	66	Cataract	OS	None	√			
3	Male	84	Cataract	OS	None	√			
4	Female	68	Cataract	OD	None		√		
5	Male	77	Cataract	OD	None		√		
6	Male	66	Cataract	OS	None		√		
7	Female	79	Cataract	OD	HTN		√		
8	Female	71	Cataract	OD	None			√	
9	Male	72	Cataract	OD	None			√	
10	Female	74	Cataract	OD	None			√	
11	Female	69	Cataract	OD	None			√	
12	Female	58	Cataract	OS	None				√
13	Male	60	Cataract	OD	None				√
14	Female	75	Cataract	OS	None				√
15	Female	55	Cataract	OD	HTN				√
Glaucoma									
1	Female	67	OAG	OD	HTN	√			
2	Male	71	OAG	OD	HTN	√			
3	Male	84	OAG	OD	HTN	√			
4	Male	61	OAG	OD	None		√		
5	Female	81	OAG	OS	HTN		√		
6	Female	80	OAG	OD	HTN		√		
7	Female	74	OAG	OD	HTN			√	
8	Female	75	OAG	OD	HTN			√	
9	Female	68	OAG	OD	HTN			√	
10	Male	43	OAG	OD	HTN				√
11	Male	61	OAG	OS	None				√
12	Female	71	OAG	OD	None				√
13	Male	69	OAG	OS	None				√
14	Male	72	OAG	OD	HTN				√

*Performed using both AH and LC samples from each participant. †RT-PCR was performed using both AH and LC samples for each participant and miRNA qRT-PCR was performed using LC samples. AH, aqueous humor; LC, lens capsule; OAG, open-angle glaucoma; HTN, hypertension; OS, oculus sinister; OD, oculus dexter; PCR, polymerase chain reaction; qRT-PCR, quantitative real-time PCR; RT-PCR, Reverse Transcription-PCR; miRNA, microRNA.

### 3.1 miRNA PCR array in AH and LC

**Figure 1:**
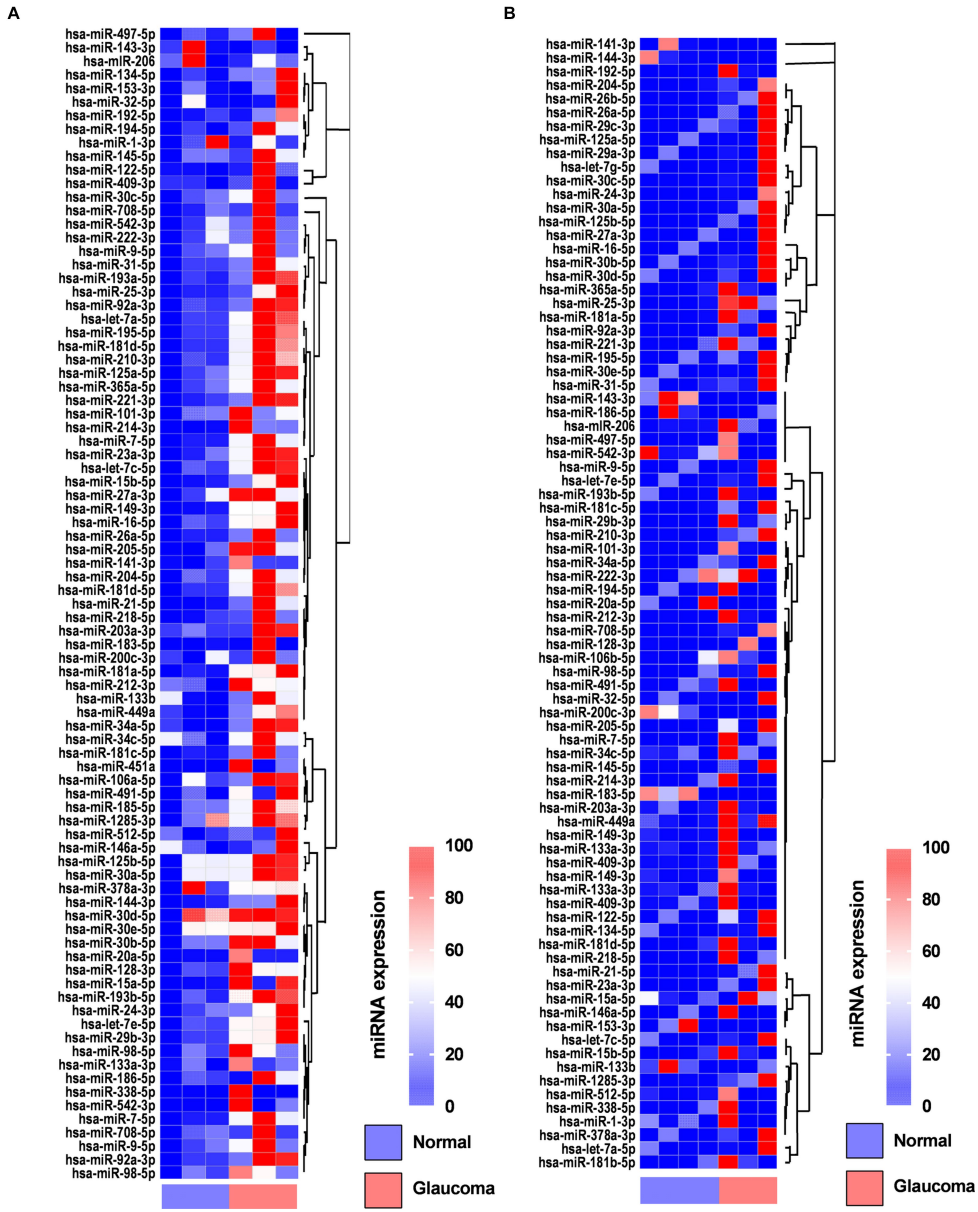
Heatmaps showing the regulated genes in the aqueous humor **(A)** and lens capsule **(B)** of patients with glaucoma and control participants using the miScript miRNA PCR array. Log2 values of 84 genes in the glaucoma group compared with the control group are represented. Colors reflect the magnitude of gene expression. Heatmaps are generated using the online Qiagen Data Analysis Center (https://geneglobe.qiagen.com/kr/analyze/).

In the AH, 19 miRNAs showed significantly differential expression between the glaucoma and control groups (Figure 1A). All 19 miRNAs (hsa-let-7a-5p, hsa-miR-125a-5p, hsa-miR-1285-3p, hsa-miR-181d-5p, hsa-miR-185-5p, hsa-miR-192-5p, hsa-miR-193a-5p, hsa-miR-194-5p, hsa-miR-195-5p, hsa-miR-210-3p, hsa-miR-221-3p, hsa-miR-222-3p, hsa-miR-25-3p, hsa-miR-31-5p, hsa-miR-365-3p, hsa-miR-512-5p, hsa-miR-542-3p, hsa-miR-9-5p, and hsa-miR-92a-5p) were significantly upregulated in the glaucoma group (*p* < 0.05; Table 2). The results of miRNA PCR array analysis of AH samples are shown in Supplementary Table S1.

**Table 2:** List of differentially expressed miRNAs from miRNA PCR arrays using aqueous humor (AH) and lens capsule (LC) samples of patients with glaucoma with statistical significance, corresponding C_T_ values, and fold change data.

miRNA	AVG ΔC_T_		2^-ΔCt^		Fold change	*p* value
	Glaucoma	Control	Glaucoma	Control		
AH						
hsa-let-7a-5p	−9.64	−7.65	799.71	200.85	3.98	0.021
hsa-miR-125a-5p	−10.66	−7.99	1618.00	254.23	6.36	0.013
hsa-miR-1285-3p	−3.33	0.5	10.03	0.71	14.16	0.024
hsa-miR-181d-5p	−5.66	−2.16	50.56	4.46	11.34	0.017
hsa-miR-185-5p	−4.5	−1.66	22.58	3.16	7.14	0.012
hsa-miR-192-5p	−4.33	0.67	20.11	0.63	31.93	0.018
hsa-miR-193a-5p	−5.99	−3.33	63.70	10.08	6.32	0.005
hsa-miR-194-5p	−2.33	3.87	5.03	0.07	73.69	0.025
hsa-miR-195-5p	−6.66	−3.99	101.13	15.93	6.35	0.017
hsa-miR-210-3p	−5.67	−1.99	50.91	3.98	12.79	0.017
hsa-miR-221-3p	−5.32	−2.33	39.85	5.02	7.94	0.025
hsa-miR-222-3p	−5.82	−3	56.36	7.98	7.06	0.016
hsa-miR-25-3p	−6.99	−3.66	127.41	12.64	10.08	0.000
hsa-miR-31-5p	−4.82	−3	28.31	7.98	3.55	0.002
hsa-miR-365-3p	−5.67	−3.33	50.80	10.08	5.04	0.018
hsa-miR-512-5p	−3.32	4.18	10.01	0.06	181.44	0.017
hsa-miR-542-3p	1.01	4.82	0.50	0.04	14.06	0.006
hsa-miR-9-5p	−0.65	1.68	1.57	0.31	5.02	0.046
hsa-miR-92a-5p	−9.99	−6.33	1019.28	80.26	12.7	0.000
LC						
hsa-let-7e-5p	−2.83	−0.74	7.093	1.6753	4.23	0.001226
hsa-miR-193a-5p	0.82	2.36	0.5661	0.1942	2.91	0.043613
hsa-miR-222-3p	2.71	4.51	0.1525	0.0438	3.48	0.027515

The miRNAs that showed significantly differential expression in the miRNA PCR array in both AH and LC samples of patients with glaucoma were miR-193a-5p and miR-222-3p. miRNA, microRNA; AH, aqueous humor; LC, lens capsule; ΔC_T_, delta C_T_; AVG ΔC_T_, average ΔC_T_; ΔC_T_ = C_T_ ([target gene] – C_T_ [reference gene]).

In the LC, three miRNAs showed significantly differential expression between the glaucoma and control groups (Figure 1B). All three miRNAs (hsa-let-7e-5p, hsa-miR-193a-5p, and has-miR-222-3p) were significantly upregulated in the glaucoma group (*p* < 0.05; Table 2). The results of miRNA PCR array analysis of LC samples are shown in Supplementary Table S2.

Among the miRNAs evaluated, hsa-miR-193a-5p and hsa-miR-222-3p were significantly upregulated in both AH and LC samples of the glaucoma group.

### 3.2 Quantitative real-time PCR in both AH and LC

**Figure 2:**
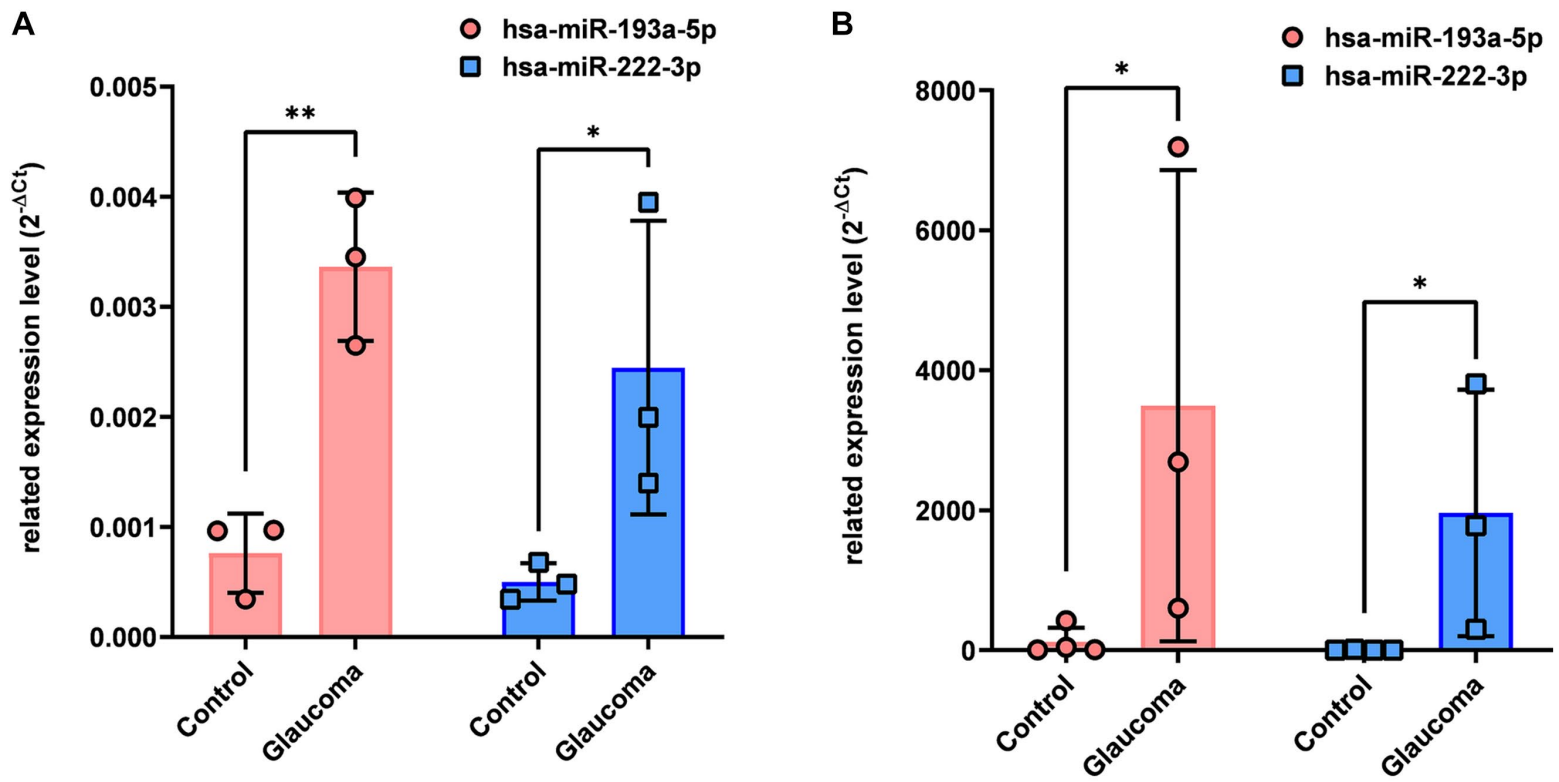
Differential expression of two microRNAs (hsa-miR-193a-5p, hsa-miR-222-3p) in aqueous humor **(A)** and lens capsule **(B)** samples of control participants and patients with glaucoma. Total RNA was isolated and expression of miRNAs for hsa-miR-193a-5p and has-miR-222-3p were analyzed using quantitative real-time PCR. Each sample was analyzed in replicate. Values on the y-axis are reported as 2^-ΔCt^. *p < 0.05, **p < 0.01 relative to the control group. Data are expressed as mean ± standard deviation. miRNA, microRNA.

qRT-PCR analysis revealed that hsa-miR-193a-5p and hsa-miR-222-3p, which were determined to be significantly upregulated in the AH and LC samples of patients with glaucoma using miRNA PCR array, were also significantly upregulated in the AH and LC samples of patients (*p* < 0.05, Figure 2). As this analysis was done from the AH and LC samples from the identical subjects, the correlation of each miRNA level between AH and LC was performed. The level of hsa-miR-193a-5p of the AH sample and that of the LC sample showed a positive correlation (*R* = 0.886, *p* = 0.009), and the level of hsa-miR-222-3p of the AH sample and that of the LC sample also showed a positive correlation (*R* = 0.754, *p* = 0.042).

### 3.3 Bioinformatics

We performed *in silico* target annotation analysis between 770 glaucoma-associated genes received from DisGeNET 7.0 database (Concept Unique Identifier “C0017601” was queried) and two upregulated miRNAs (hsa-miR-193a-5p and hsa-miR-222-3p). Target annotation analysis revealed 57 validated miRNA:gene pairs (Supplementary Table S3) and 69 top 10% predicted miRNA:gene pairs obtained with the highest probability (Supplementary Table S4). Among the experimentally validated miRNA-gene regulatory interactions, eight miRNA:gene pairs were validated based on the experiments known as strong evidences (Table 3).

**Table 3:** List of experimentally validated miRNA:gene pairs between 770 glaucoma-associated genes received from the DisGeNET 7.0 database and two miRNAs (hsa-miR-193a-5p and hsa-miR-222-3p) using multiMiR 1.10.0 package.

Target gene name	
hsa-miR-193a-5p	hsa-miR-222-3p
*MTOR*	*CDKN1B*
*WT1*	*ETS1*
	*MMP1*
	*PTEN*
	*SOD2*
	*TIMP3*

The pairs here only include those validated with strong evidence, including luciferase reporter assay, western blot, and RT-PCR. The whole list is listed in the Supplementary Table S3. MTOR; mammalian target of rapamycin, WT1; Wilms tumor protein, CDKN1B; cyclin dependent kinase inhibitor 1B, ETS1; ETS proto-oncogene 1 (transcription factor), MMP-1; matrix metalloproteinase-1, PTEN; phosphatase and tensin homolog, SOD2; superoxide dismutase 2, TIMP3; TIMP.

**Figure 3:**
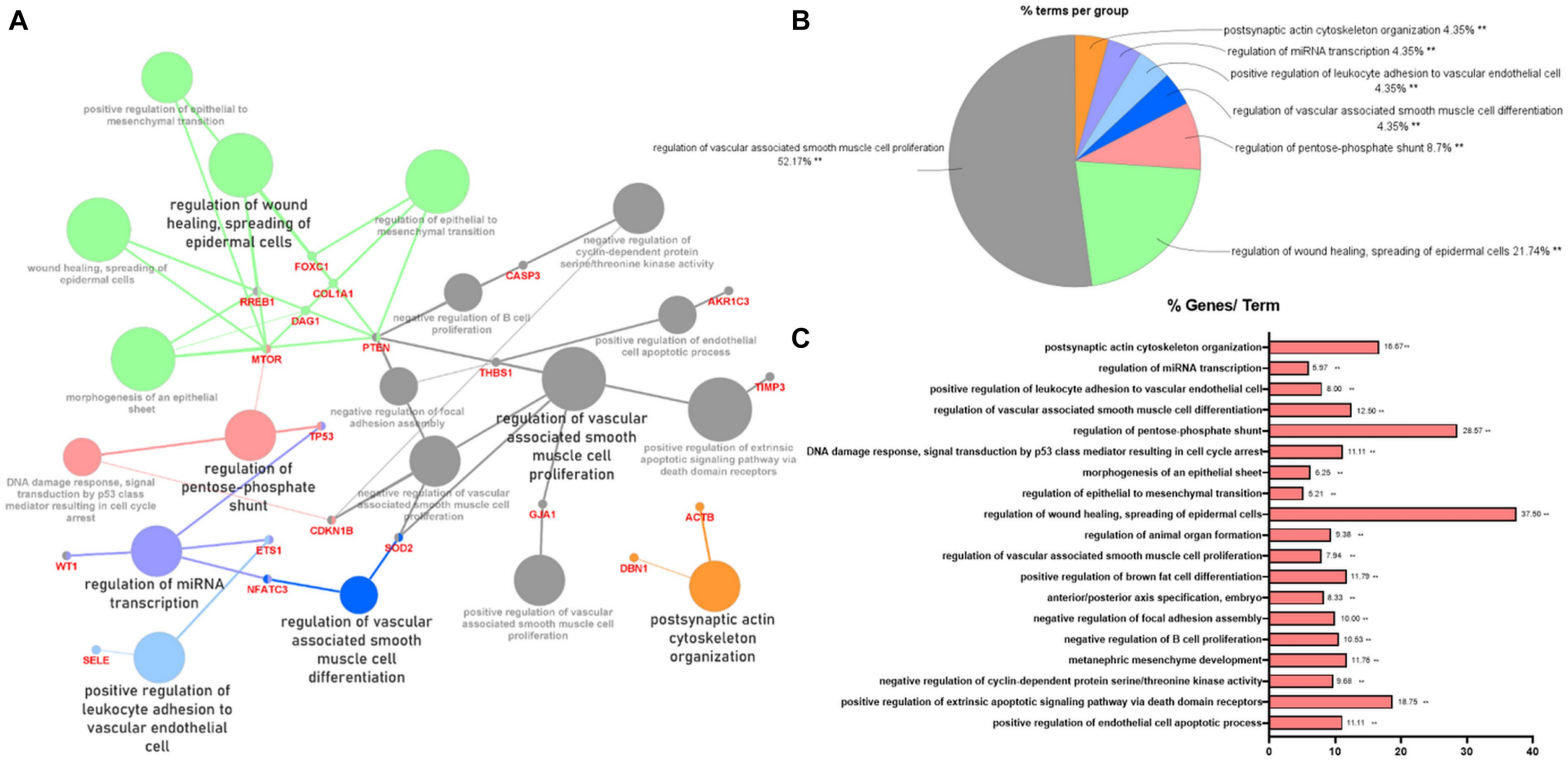
Gene ontology enrichment analysis with significant pathways. **(A)** Enrichment analysis showing interactions of target genes of miR-193a-5p and miR-222-3p. All interactions were statistically significant. The color indicates the grouping of the terms. **(B)** A pie chart of biological processes associated with the query genes. Frequency of each group is shown based on percentage. **(C)** Category plot displays GO/pathway terms that were related to target genes. The bars represent the percentage of genes associated with the terms. The number of genes per term is shown as bar label.

To indicate the biological processes in which miRNA-regulated genes are involved, gene ontology (GO) enrichment analysis with significant pathways was performed using 57 validated target genes. GO enrichment analysis using ClueGO of Cytoscape revealed that 23 terms, including “regulation of wound healing, spreading of epidermal cells,” “regulation of extrinsic apoptotic signaling pathway via death domain receptors,” and “positive and negative regulation of vascular-associated smooth muscle cell proliferation,” were associated with the validated target genes of the two miRNAs (Figure 3A). Supplementary Table S5 summarizes the related terms and genes included in each term. The top three groups were as follows: regulation of vascular-associated smooth muscle cell proliferation (52.17%), regulation of wound healing, spreading of epidermal cells (21.74%), and regulation of pentose-phosphate shunt (8.7%; Figure 3B). Figure 3C shows the percentage of the number of validated target genes associated with each term out of the total number of genes associated with that term (%Genes/Term). The % Genes/Term was the highest in the term “Regulation of wound healing, spreading of epidermal cells.”

Bioinformatics analysis revealed that the two miRNAs (hsa-miR-193a-5p and hsa-miR-222-3p), apoptosis signaling pathways, and glaucoma were correlated (Supplementary Figure S1). These two miRNAs were associated with survival or apoptosis-related factors, such as *TGFB1*, *PI3K*, *BAX*, *BCL2*, *TP53*, *PTEN*, *CASP3*, *AKT*, and cyclin D1.

### 3.4 Expression level for the selected target gene (*PTEN*) in AH and LC

**Figure 4:**
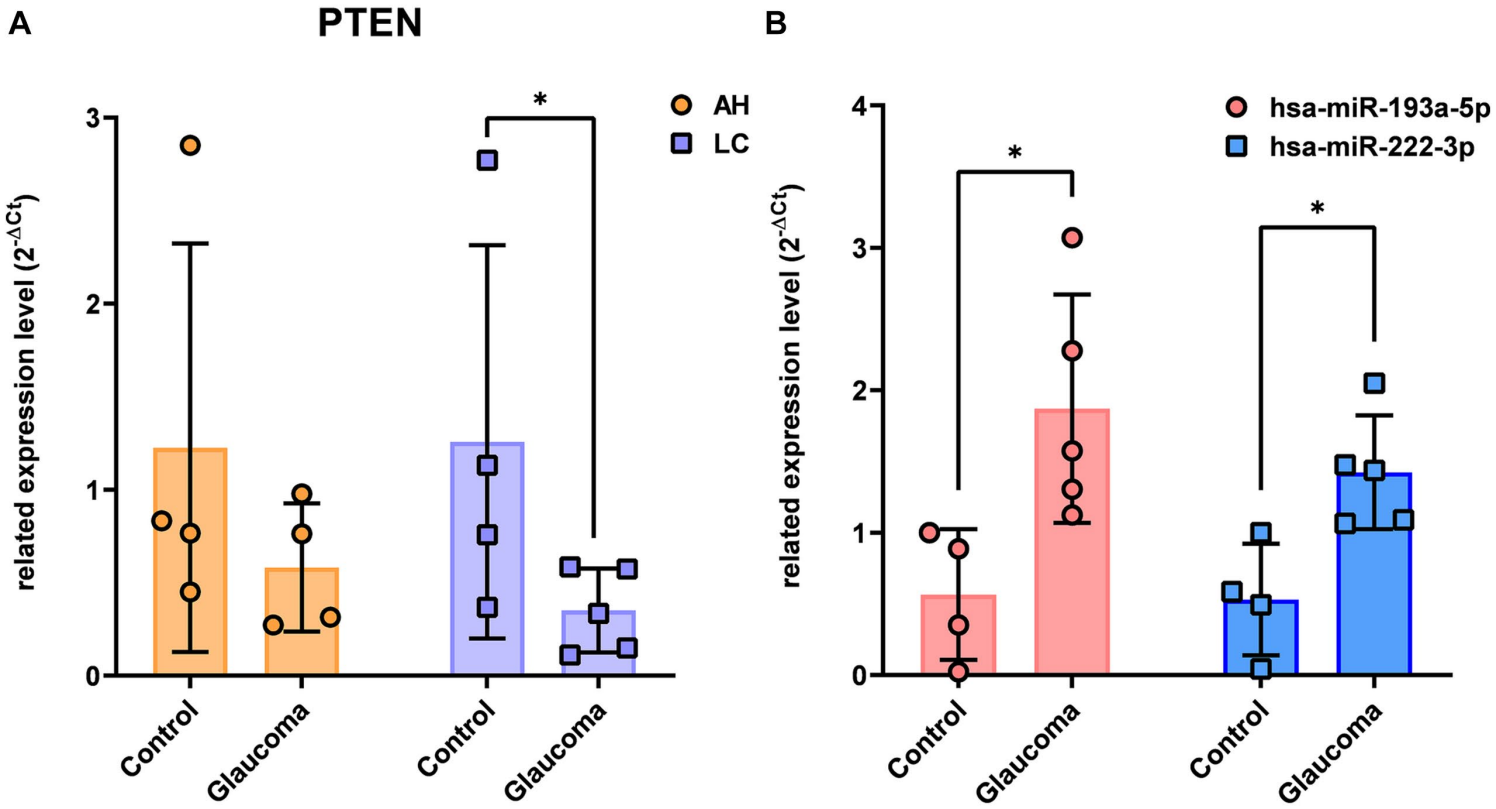
The expression level of *PTEN* in the aqueous humor and lens capsule samples of control participants and patients with glaucoma **(A)** and the differential expression of two microRNAs, hsa-miR-193a-5p and hsa-miR-222-3p, in the lens capsule samples of control participants and patients with glaucoma **(B)**. The expression of *PTEN* was analyzed using RT-PCR. Total RNA was isolated, and expression of miRNAs for hsa-miR-193a-5p and has-miR-222-3p were analyzed using qRT-PCR. Each sample was analyzed in a replicate. Values on the y-axis are reported as 2^-ΔCt^. *p < 0.05, **p < 0.01 relative to the control group. Data are expressed as mean ± standard deviation. *PTEN*, phosphatase and tensin homolog; miRNA, microRNA; AH, aqueous humor; LC, lens capsule; RT-PCR, Reverse Transcription-PCR; qRT-PCR, quantitative real-time PCR.

There were no overlapping target genes between the two miRNAs which are validated by experiments corresponding to strong evidence. Among the 8 target genes listed in Table 3, the further literature review revealed a reported association between *MTOR* and hsa-miR-222-3p in pancreatic cancer (22), *ETS1* and hsa-miR-193a-5p in gastric cancer (23), and *PTEN* and hsa-miR-193a-5p in Alzheimer’s disease (24). We selected *PTEN* further to analyze mRNA and miRNA correlations in patient samples. AH and LC samples from a separate cohort (four control subjects and five glaucoma patients) were used for RT-PCR of *PTEN* and qRT-PCR of hsa-miR-193a-5p and hsa-miR-222-3p. Only *PTEN* could be analyzed because of the small amount of RNA in AH samples, whereas *PTEN* and the two miRNAs could be analyzed in LC samples. In one AH sample from the glaucoma group, the RNA quality was unsuitable for the analysis. Consequently, LC samples of four control subjects and five patients with glaucoma, and AH samples of four control subjects and four patients with glaucoma were analyzed. The results showed significant upregulation of the two miRNAs in the LC samples of glaucoma group, concurrently with significant downregulation of *PTEN* in the LC samples of glaucoma group (*p* < 0.05; Figure 4). *PTEN* levels in the AH samples did not differ significantly between the control and glaucoma groups.

### 3.5 *In vitro* validation of the regulatory roles of the miRNAs (hsa-miR-193a-5p and hsa-miR-222-3p) on the selected target gene (*PTEN*) in a glaucomatous cell model

**Figure 5:**
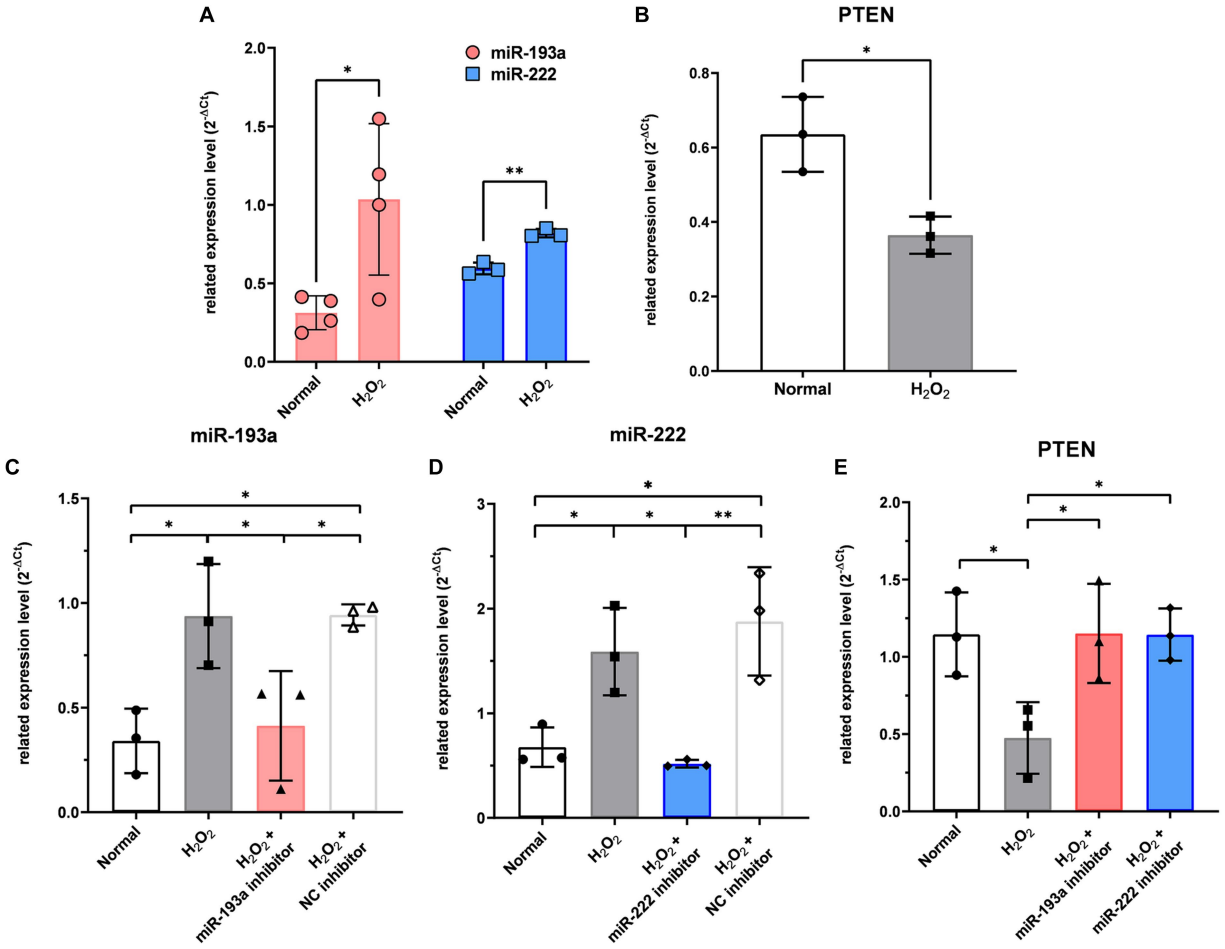
The expression level of the two differentially expressed microRNAs (miR-193a-5p and miR-222-3p) **(A)** and *PTEN* **(B)** in the retinal ganglion cell (RGC) model under oxidative stress using H_2_O_2_. Transfection of miR-193a inhibitor **(C)** and miR-222 inhibitor **(D)** to RGC showed regulation of expression of the miRNAs. Negative control (NC) inhibitor had no effect on the expression of the miRNAs. The two miRNA inhibitors also up-regulated the expression of *PTEN*, which was reduced by H_2_O_2_ **(E)**. Values on the y-axis are reported as 2^-ΔCt^. *p < 0.05. Data are expressed as mean ± standard deviation.

In RGCs exposed to H_2_O_2_-induced oxidative injury, the expression levels of hsa-miR-193a-5p and hsa-miR-222-3p significantly increased (Figure 5A), while that of *PTEN* significantly decreased (Figure 5B). Treatment with the hsa-miR-193a-5p inhibitor resulted in a decrease in hsa-miR-193a-5p expression (Figure 5C) and an increase in *PTEN* expression (Figure 5E), a pattern replicated with the hsa-miR-222-3p inhibitor (Figures 5D,E). These findings imply the regulatory roles of these miRNAs on PTEN in RGCs under oxidative stress.

## 4 Discussion

This study analyzed the apoptosis-related miRNA profiles of both AH and LC samples in patients with glaucoma and control participants. The expression levels of hsa-miR-193a-5p and hsa-miR-222-3p in both AH and LC samples of patients with glaucoma were upregulated when compared with those in both AH and LC samples of controls. Additionally, this study examined the role of these miRNAs in glaucoma using bioinformatics tools and identified their target genes and associated biological processes (survival and apoptotic signaling pathways). Then, based on a literature review, we selected a putative gene (*PTEN*) to determine its differential expression in AH and LC samples from patients with glaucoma. We further validated the differential expression of the target miRNAs and the target gene through *in vitro* experiments, demonstrating the regulatory role of these miRNAs-*PTEN* axes. This is the first study to use both AH and LC samples for miRNA profiling in patients with glaucoma and reveal the related biological pathways using bioinformatics tools that are experimentally validated.

Several miRNA studies on glaucoma have used experimental animal/cell models and AH or blood samples from patients to elucidate the pathological mechanisms of glaucoma, although no specific consensus has been achieved. Experimental models of glaucoma used in miRNA studies include animal models and damaged RGCs. One study investigated the changes in miRNA expression in RGCs in the laser-induced ocular hypertension rat model and identified potential neuroprotective candidates for RGCs (miR-194 and miR-664-2 inhibitors) (25). Other studies have reported that various miRNAs, such as miR-141-3p, miR-141-5p, miR-21a-5p, and miR-93-5p, which inhibited RGC apoptosis and promoted RGC survival in the N-methyl-D-aspartic acid (NMDA)-induced glaucomatous mouse model, are potential therapeutic targets (26–29). Studies have also been performed to identify potential related biological processes. The overexpression of miR-124b protects RGC function, activates autophagy, and modulates survival signaling pathways, such as *mTOR* and *AKT*, in the surgically-induced glaucomatous neurodegeneration mouse model (30). There have been several studies that analyzed miRNAs expression in patients with glaucoma using samples, such as blood (plasma or serum) or AH. A recent review summarized the findings of various miRNA studies using samples from patients with open-angle glaucoma and suggested that miR-143-3p, miR-125b-5p, and miR-1260b are potential therapeutic targets for glaucoma (31). However, the authors also suggested the lack of overlapping findings among the miRNA profiling studies conducted using the AH (13, 20, 32–38) and other samples, including blood (plasma/serum) and tears of patients with primary open-angle glaucoma (13, 32, 39, 40). The results of miRNA profiling studies using intraocular samples of patients with glaucoma are summarized in Supplementary Table S6. Blood samples can be obtained through minimally invasive procedures and collected at frequent intervals. Recent studies showed that miRNAs in serum samples are biomarkers that serve as valuable indicators of systemic and ocular diseases (41–44). However, AH samples can more directly reflect eye-specific conditions than serum samples. Meanwhile, LC can directly reflect the condition of the anterior chamber, including the TM tissue. miRNA profiling of the LC samples of patients with open-angle glaucoma has not been previously performed. Hence, this study aimed to analyze the miRNA expression profile in both AH and LC.

This study demonstrated that the two miRNAs hsa-miR-193-5p and hsa-miR-222-3p were upregulated in both AH and LC in patients with open-angle glaucoma. Upregulation of has-miR-222-3p in AH of patients with normal tension glaucoma has recently been reported (33), the dysregulation of hsa-miR-193-5p has not been previously reported in patients with glaucoma. The roles of hsa-miR-193-5p and hsa-miR-222-3p in apoptotic pathways have been extensively studied (40, 45–48). Consistently, Laverne bioinformatics analysis also revealed the association between these two miRNAs and the apoptotic pathway in this study (Supplementary Figure S1). The roles of hsa-miR-193-5p and hsa-miR-222-3p have mostly been studied in the context of cancer biology, including angiogenesis (49, 50) and inflammation (51, 52). miR-193a-5p promotes apoptosis and consequently inhibits colorectal cancer cell survival through the miR-193a-5p/PIK3R3/AKT axis (53). One study reported that miR-193a-5p mimics decrease cancer cell viability and suppress intracellular mechanisms (54). miR-222-3p interacted with circular RNA (circRNA) to inhibit cancer cell development (47). Circ_HIPK3, a circRNA, was reported to protect neuronal cells against apoptosis by inhibiting the miR-222-3p/DUSP19 axis (55). miR-19b/221/222 induces endothelial cell dysfunction and cellular apoptosis through the suppression of proliferator-activated receptor γ coactivator 1α (PGC-1α) (56). Additionally, a previous study examined the roles of miR-222-3p in human lens epithelial cells of the *in vitro* cataract model and reported that the circRNA-mediated inhibition of miR-222-3p mitigates cell damage (57). These previous findings demonstrate that miR-222-3p promotes cell damage by inducing oxidative stress and apoptosis, which are important pathological components of glaucoma (58–61). Previous studies have also reported the association between miRNAs and oxidative stress/apoptosis in glaucoma (12, 40). In summary, our findings are consistent with the previous studies showing that miR-193a and miR-222 tend to induce oxidative stress and apoptosis.

In this study, we selected *PTEN* among the validated target genes to analyze the differences in its expression levels in AH and LC samples between control subjects and patients with glaucoma. Because the amount of AH and LC samples was insufficient to analyze multiple target genes simultaneously with miRNAs, we selected one target gene. Expression of *PTEN* was significantly downregulated in LC samples from patients with glaucoma, whereas it was not statistically significant in AH samples. *PTEN* is involved in multiple cellular processes, including cell proliferation, apoptosis, and migration, and cell-ECM interaction and signaling (62). The beneficial effects of *PTEN* inhibition, including neuroprotective effects, have been reported mainly through the upregulation of the Akt signaling pathway (63); however, beneficial effects of *PTEN* upregulation through treatment with human *PTEN* in neuronal survival have also been reported (64). Analysis of signaling pathway activation using TM tissues obtained from OAG donors revealed significant downregulation of the *PTEN* pathway, which is thought to be associated with apoptosis in the TM (65). In addition, the critical role of *PTEN* in regulating ECM remodeling in tissues is known and a decrease in *PTEN* levels has been reported in many fibrotic diseases (66). It has been reported that a reduction in *PTEN* levels in TM cells contributes to fibrosis of TM by inducing ECM deposition, which may lead to dysfunction of AH drainage (67). In this study, *PTEN* levels were significantly downregulated in LC samples from patients with glaucoma, although this was not significant in AH samples. Since the interactions between *PTEN* and both miRs-193-5p and − 222-3p are known (24, 68), it can be hypothesized that the upregulation of miRs-193a and − 222 and subsequent downregulation of *PTEN* may have a potential role in OAG. We further validated the differential expression of these miRNAs and *PTEN* in the oxidative-injured RGC model, one of the glaucomatous cell models, and also revealed the regulatory roles of these miRNAs (miRs-193-5p and − 222-3p) on *PTEN* for the first time. The exact roles of these miRNAs/*PTEN* axes in the pathogenesis of glaucoma needs to be elucidated through experimental validation in future studies.

In addition to the two miRNAs that were upregulated in both AH and LC samples, we also identified miRNAs with inconsistent upregulation in AH and LC in this study. The upregulated miRNAs in the AH and LC of patients with glaucoma included other 17 miRNAs (hsa-let-7a-5p, hsa-miR-125a-5p, hsa-miR-1285-3p, hsa-miR-181d-5p, hsa-miR-185-5p, hsa-miR-192-5p, hsa-miR-194-5p, hsa-miR-195-5p, hsa-miR-210-3p, hsa-miR-221-3p, hsa-miR-25-3p, hsa-miR-31-5p, hsa-miR-365-3p, hsa-miR-512-5p, hsa-miR-542-3p, hsa-miR-9-5p, and hsa-miR-92a-5p), and hsa-let-7e-5p, respectively. Previous studies have reported the upregulation of hsa-let-7a-5p and hsa-miR-192-5p in the AH of patients with normal tension glaucoma (33, 38), hsa-miR-210-3p in the serum of patients with primary open-angle glaucoma (POAG) (39), hsa-miR-221-3p in the AH of patients with POAG (32), and hsa-miR-9-5p in the peripheral blood mononuclear cells of patients with pseudoexfoliation glaucoma (14). The hsa-miR-125a-5p levels are upregulated in non-glaucomatous human TM cells in response to cyclic mechanical stretch, and this is suggested to be correlated with the pathogenesis of glaucoma (69). On the other hand, the expression of miR-181d-5p in RGC was downregulated in the glaucomatous mouse model (25), and that of miR-25 was downregulated in glaucomatous rat retina (70). These discrepancies can be attributed to differences in the samples used in different studies (animal models in previous studies vs. AH or LC samples of patients in this study). The roles of hsa-miR-185-5p, hsa-miR-194-5p, hsa-miR-195-5p, hsa-miR-31-5p, hsa-miR-92a-5p, and let-7e-5p in glaucoma have not been reported.

This study has some limitations. First, small numbers of participants were included in this study and separate samples were used for each analysis. As the amount of AH was insufficient to be used simultaneously for miRNA PCR array and qRT-PCR analyses, separate samples were used for each analysis. In addition, we initially planned to use the same participants’ AH and LC samples for the miRNA PCR array. However, after miRNA extraction and quality control processes, the AH and LC samples available for miRNA PCR array were derived from separate subjects. Consequently, miRNA PCR array was performed using AH and LC samples collected from separate patient groups. Instead, qRT-PCR was performed using AH and LC samples collected from the same eye of the same subject. Second, further studies using animal models were not conducted. Therefore, further research is needed to determine the mechanisms and effects of the identified miRNAs on glaucoma. Additionally, *in vitro* studies using TM tissue will be beneficial to investigate the relationship between these miRNAs and apoptosis of TM cells that could not be conducted in this study. Finally, it was not possible to assess the association between miRNA expression levels and clinical factors such as the severity of glaucoma owing to the small number of participants in each miRNA analysis. Despite these limitations, this is the first study to report the miRNA profiles of both AH and LC samples in patients with glaucoma. The miRNA profiles found in this study would reflect the intraocular status better than those found in previous studies conducted using only AH of patients with glaucoma. Circulating miRNAs can be considered good potential biomarkers in many diseases (42). We analyzed apoptosis-related miRNAs in AH and LC samples and found two common miRNAs that showed significant differential expression in both AH and LC and were associated with *PTEN*. These miRNAs and *PTEN* have been shown to be associated with apoptosis/survival in other diseases. Therefore, although the role of these miRNAs as biomarkers for glaucoma could not be validated in this study, we propose that these miRNAs and *PTEN*, or their modulation, may represent potential diagnostic biomarkers or therapeutic strategies in glaucoma.

To the best of our knowledge, this is the first study to elucidate the expression profiles of apoptosis-related miRNAs in AH and LC samples of patients with open-angle glaucoma. Two miRNAs, hsa-miR-193a-5p and hsa-miR-222-3p, were upregulated in both AH and LC samples of patients with glaucoma, and their common putative gene was *PTEN*, which was downregulated in both AH and LC samples of patients with glaucoma. The functions of these two miRNAs and *PTEN* are related to apoptosis and oxidative stress, which are involved in the pathogenesis of open-angle glaucoma. Therefore, these miRNAs may serve as novel biomarkers or therapeutic targets in open-angle glaucoma.

## Data availability statement

Original datasets are available in a publicly accessible repository: ArrayExpress. The original contributions presented in the study are publicly available. This data can be found here: [https://www.ebi.ac.uk/fg/annotare/edit/18328/] [accession number: E-MTAB-13771].

## Ethics statement

The studies involving humans were approved by the institutional review board (IRB) of Hanyang University Guri Hospital. The studies were conducted in accordance with the local legislation and institutional requirements. The participants provided their written informed consent to participate in this study.

## Author contributions

HY: Formal analysis, Investigation, Methodology, Project administration, Visualization, Writing – original draft. EH: Funding acquisition, Investigation, Project administration, Validation, Writing – original draft. JK: Data curation, Software, Writing – review & editing. YL: Validation, Writing – review & editing. WL: Formal analysis, Writing – review & editing, Funding acquisition. MK: Resources, Writing – review & editing. HC: Conceptualization, Resources, Writing – review & editing. YS: Conceptualization, Data curation, Funding acquisition, Supervision, Writing – review & editing. MS: Conceptualization, Data curation, Funding acquisition, Resources, Supervision, Writing – review & editing.

## Footnotes

^1^ https://geneglobe.qiagen.com/

^2^ https://geneglobe.qiagen.com/kr/analyze/

^3^ https://www.r-project.org/

^4^ https://www.disgenet.org/

^5^ https://bioconductor.org/packages/release/bioc/html/multiMiR.html

^6^ http://www.novusbio.com/explorer/
