# Supplementary material for: Expression of microRNAs related to apoptosis in the aqueous humor and lens capsule of patients with glaucoma
Source: Front Med (Lausanne). 2024 Feb 14;11:1288854. doi: 10.3389/fmed.2024.1288854 (PMC10917207; doi:10.3389/fmed.2024.1288854)
Supplement: Supplementary file 1 [file Data_Sheet_1.pdf]

Expression of microRNAs related to apoptosis in the aqueous humor and lens capsule of patients with glaucoma

Supplementary Material

Supplementary Figure S1. Volcano plots show different expression of miRNAs in aqueous humor (A) and lens capsule (B) samples from control subjects versus patients with glaucoma. The dashed horizontal line indicates statistical significance threshold ($p < 0.05$).

miRNA, microRNA; AH, aqueous humor; LC, lens capsule; X-axis: log_2 fold change, Y-axis: -log10(p-value).

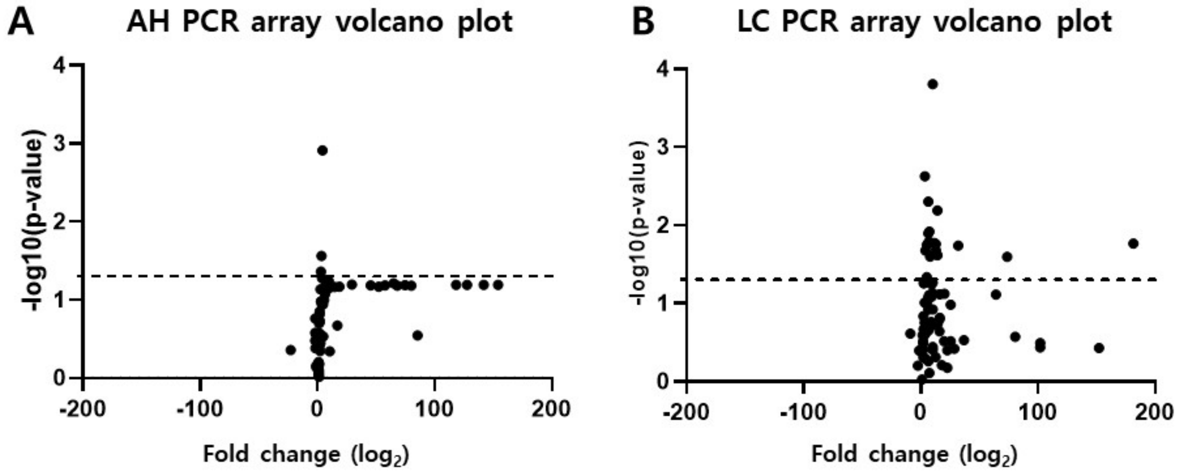

Supplementary Figure S2. Gene relationships between two miRNAs (hsa-miR-193a-5p and hsa-miR-222-3p), glaucoma, and apoptosis were determined using Novus Biologicals bioinformatic analysis.

TGF-β, transforming growth factor-beta – beta, PI3K; phosphoinositide 3-kinase; BAX; BCL2 Associated X, BCL-2, B-cell lymphoma 2; TP53, tumor protein 53; PTEN, phosphatase and tensin homolog; CASP3, caspase 3; AKT, protein kinase B

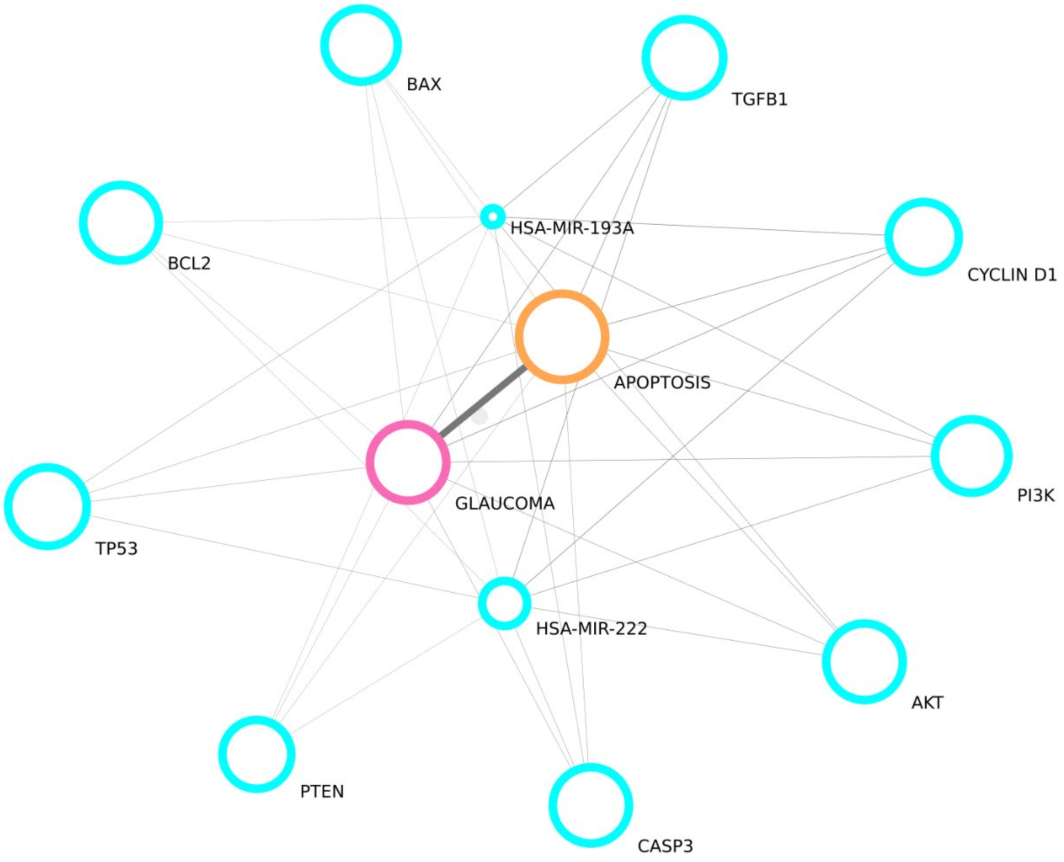

Supplementary Table S1. miScript PCR array data of the expression of 84 miRNAs in the aqueous humor of patients with glaucoma compared with control participants. Nineteen miRNAs were significantly upregulated in the aqueous humor of patients with glaucoma.

Gene	Fold Change	p-value	Gene	Fold Change	p-value
hsa-let-7a-5p	3.98	0.021192	hsa-miR-181a-5p	3.96	0.180265
sa-miR-125a-5p	6.36	0.012779	hsa-miR-181b-5p	3.95	0.220783
sa-miR-1285-3p	14.16	0.024332	hsa-miR-181c-5p	2.27	0.214142
sa-miR-181d-5p	11.34	0.016923	hsa-miR-183-5p	10.08	0.361842
sa-miR-185-5p	7.14	0.012074	hsa-miR-186-3p	101.83	0.366358
sa-miR-192-5p	31.93	0.018258	hsa-miR-193b-3p	10.08	0.119453
sa-miR-193a-5p	6.32	0.004974	hsa-miR-200c-3p	101.83	0.321923
sa-miR-194-5p	73.69	0.025376	hsa-miR-203a-3p	80.63	0.268887
sa-miR-195-5p	6.35	0.016969	hsa-miR-204-5p	3.97	0.241807
sa-miR-210-3p	12.79	0.017496	hsa-miR-205-5p	15.89	0.228553
sa-miR-221-3p	7.94	0.025228	hsa-miR-206	14.25	0.182202
sa-miR-222-3p	7.06	0.016282	hsa-miR-20a-5p	1.42	0.256232
sa-miR-25-3p	10.08	0.000157	hsa-miR-21-5p	15.85	0.160260
sa-miR-31-5p	3.55	0.002367	hsa-miR-212-3p	12.61	0.488837
sa-miR-365b-3p	5.04	0.017937	hsa-miR-214-3p	10.03	0.187989
sa-miR-512-5p	181.44	0.017227	hsa-miR-218-5p	7.13	0.206144
sa-miR-542-3p	14.06	0.006478	hsa-miR-23a-3p	10.13	0.073698
hsa-miR-9-5p	5.02	0.046337	hsa-miR-24-3p	7.29	0.053158
sa-miR-92a-3p	12.7	0.000209	hsa-miR-26a-5p	2	0.146547
hsa-let-7c-5p	7.96	0.079280	hsa-miR-26b-5p	2.49	0.055739
hsa-let-7e-5p	5.02	0.101360	hsa-miR-27a-3p	7.06	0.078761
hsa-let-7g-5p	2.51	0.054689	hsa-miR-29a-3p	10.08	0.054360
hsa-miR-1-3p	25.4	0.104088	hsa-miR-29b-3p	8.96	0.083016
sa-miR-101-3p	16.04	0.153358	hsa-miR-29c-3p	10.06	0.053907
sa-miR-106b-5p	1	0.941548	hsa-miR-30a-5p	6.35	0.083900
sa-miR-122-5p	6.32	0.550510	hsa-miR-30b-5p	1.98	0.307253
sa-miR-128-3p	3.17	0.176594	hsa-miR-30c-5p	2	0.390500
sa-miR-125b-5p	5.03	0.094698	hsa-miR-30d-5p	6.35	0.222801
sa-miR-133a-3p	2.22	0.492481	hsa-miR-30e-5p	6.28	0.211443
hsa-miR-133b	22.63	0.400553	hsa-miR-32-5p	152.22	0.373662
sa-miR-134-5p	25.46	0.305653	hsa-miR-338-3p	0.71	0.402732
sa-miR-141-3p	19.84	0.304594	hsa-miR-34a-5p	22.47	0.669752
sa-miR-143-3p	10.03	0.393392	hsa-miR-34c-5p	17.88	0.617257
sa-miR-144-3p	4.02	0.192188	hsa-miR-378a-3p	2.82	0.257799
sa-miR-145-5p	8.96	0.082678	hsa-miR-409-3p	7.14	0.780209
sa-miR-146a-5p	64	0.077404	hsa-miR-449a	28.64	0.382775
sa-miR-149-3p	20.21	0.075739	hsa-miR-451a	0.7	0.405922
sa-miR-153-3p	36.67	0.295152	hsa-miR-491-5p	3.56	0.506144
sa-miR-15a-5p	8.88	0.173553	hsa-miR-497-5p	-0.11	0.244749
sa-miR-15b-5p	16	0.076478	hsa-miR-7-5p	6.31	0.120788
sa-miR-16-5p	8.96	0.057039	hsa-miR-708-5p	3.16	0.098204
sa-miR-17-5p	-0.39	0.629614	hsa-miR-98-5p	1.99	0.325641

Supplementary Table S2. miScript PCR array data of the expression of 84 miRNAs in the lens capsule of patients with glaucoma compared with control participants. Three miRNAs were significantly upregulated in the lens capsule of patients with glaucoma.

Gene	Fold Change	p-value	Gene	Fold Change	p-value
hsa-let-7e-5p	4.23	0.001226	hsa-miR-205-5p	14.51	0.068428
sa-miR-193a-5p	2.91	0.043613	hsa-miR-206	203.13	0.064414
sa-miR-222-3p	3.48	0.027515	hsa-miR-20a-5p	462492.62	0.285591
hsa-let-7a-5p	1.81	0.153437	hsa-miR-21-5p	0.9	0.726794
hsa-let-7c-5p	1.5	0.200459	hsa-miR-210-3p	5.7	0.100167
hsa-let-7g-5p	0.93	0.624292	hsa-miR-212-3p	21108.55	0.285591
hsa-miR-1-3p	780.87	0.283843	hsa-miR-214-3p	68.09	0.065664
sa-miR-101-3p	9.6	0.057199	hsa-miR-218-5p	3933012.11	0.131280
sa-miR-106b-5p	4.71	0.114066	hsa-miR-221-3p	1.9	0.190774
sa-miR-122-5p	127.71	0.064502	hsa-miR-23a-3p	0.92	0.693937
sa-miR-125a-5p	0.57	0.422629	hsa-miR-24-3p	1.05	0.897237
sa-miR-125b-5p	1.19	0.808891	hsa-miR-25-3p	1.05	0.760677
sa-miR-128-3p	6.63	0.084099	hsa-miR-26a-5p	0.54	0.264346
sa-miR-1285-3p	52.23	0.068105	hsa-miR-26b-5p	0.56	0.172741
sa-miR-133a-3p	154.12	0.064434	hsa-miR-27a-3p	1.39	0.963427
hsa-miR-133b	125167.74	0.140413	hsa-miR-29a-3p	0.55	0.332580
sa-miR-134-5p	74.78	0.064653	hsa-miR-29b-3p	2.66	0.073130
sa-miR-141-3p	10.57	0.456387	hsa-miR-29c-3p	0.62	0.415635
sa-miR-143-3p	203.13	0.064414	hsa-miR-30a-5p	1.17	0.868670
sa-miR-144-3p	43413.1	0.463841	hsa-miR-30b-5p	1.19	0.957678
sa-miR-145-5p	29.48	0.063972	hsa-miR-30c-5p	1.1	0.800251
sa-miR-146a-5p	1687.72	0.285298	hsa-miR-30d-5p	2.26	0.453575
sa-miR-149-3p	118.39	0.064479	hsa-miR-30e-5p	2.15	0.307328
sa-miR-153-3p	27146.5	0.285590	hsa-miR-31-5p	2.56	0.358449
sa-miR-15a-5p	1158.15	0.071554	hsa-miR-32-5p	65.09	0.061216
sa-miR-15b-5p	2.03	0.270371	hsa-miR-338-3p	16.9	0.212567
sa-miR-16-5p	1.15	0.654173	hsa-miR-34a-5p	6.31	0.072093
sa-miR-17-5p	3.83	0.114816	hsa-miR-34c-5p	80.21	0.066024
sa-miR-181a-5p	1.02	0.927495	hsa-miR-365b-3p	1.51	0.670362
sa-miR-181b-5p	1.71	0.394444	hsa-miR-378a-3p	85.55	0.285586
sa-miR-181c-5p	3.19	0.104186	hsa-miR-409-3p	141.88	0.064445
sa-miR-181d-5p	2.93	0.105464	hsa-miR-449a	119.01	0.064486
sa-miR-183-5p	57.73	0.065159	hsa-miR-451a	38269.98	0.749643
sa-miR-185-5p	1.98	0.284324	hsa-miR-491-5p	6.97	0.058276
sa-miR-186-3p	210996.81	0.285591	hsa-miR-497-5p	13930.38	0.285591
sa-miR-192-5p	-0.04	0.436588	hsa-miR-512-5p	4102.53	0.142906
sa-miR-193b-3p	4.93	0.295033	hsa-miR-542-3p	203322.4	0.285591
sa-miR-194-5p	6840.52	0.279944	hsa-miR-7-5p	18.71	0.067857
sa-miR-195-5p	1.63	0.643414	hsa-miR-708-5p	1.98	0.185580
sa-miR-200c-3p	9.37	0.071841	hsa-miR-9-5p	4522.37	0.285591
sa-miR-203a-3p	45.49	0.065148	hsa-miR-92a-3p	1.78	0.140465
sa-miR-204-5p	0.83	0.275723	hsa-miR-98-5p	3.9	0.052728

Supplementary Table S3. List of experimentally validated miRNA:gene pairs found in *silico* among the two upregulated miRNAs in both the aqueous humor and lens capsule of patients with glaucoma and 770 genes associated with glaucoma.

No.	miRNA miRBase ID	Gene symbol	Database	Validation method
1.	hsa-miR-193a-5p	ARSD	mirtarbase	HITS-CLIP
2.	hsa-miR-193a-5p	BIRC6	tarbase	Degradome sequencing
3.	hsa-miR-193a-5p	CEBPD	tarbase	Degradome sequencing
4.	hsa-miR-193a-5p	COL1A1	tarbase	Degradome sequencing
5.	hsa-miR-193a-5p	CYP1B1	tarbase	Degradome sequencing
6.	**hsa-miR-193a-5p**	**MTOR**	mirtarbase	**Luciferase reporter assay, Western blot, qRT-PCR**
7.	hsa-miR-193a-5p	PLXDC2	mirtarbase	PAR-CLIP
8.	hsa-miR-193a-5p	WNK1	tarbase	Degradome sequencing
9.	**hsa-miR-193a-5p**	**WT1**	mirtarbase	Immunofluorescence, **Luciferase reporter assay, Western blot, qRT-PCR**
10.	hsa-miR-222-3p	ACTB	mirtarbase	CLASH
11.	hsa-miR-222-3p	AKR1C3	tarbase	Degradome sequencing
12.	hsa-miR-222-3p	ALDH1A1	tarbase	Degradome sequencing
13.	hsa-miR-222-3p	ATF2	tarbase	Degradome sequencing
14.	hsa-miR-222-3p	B3GALNT2	mirtarbase; tarbase	PAR-CLIP//Degradome sequencing
15.	hsa-miR-222-3p	BCOR	tarbase	Degradome sequencing
16.	hsa-miR-222-3p	BIRC6	tarbase	Degradome sequencing
17.	hsa-miR-222-3p	BUB1B	tarbase	Degradome sequencing
18.	hsa-miR-222-3p	BUB3	tarbase	Degradome sequencing
19.	hsa-miR-222-3p	CASP3	tarbase	Degradome sequencing
20.	hsa-miR-222-3p	CAV2	tarbase	Degradome sequencing
21.	**hsa-miR-222-3p**	**CDKN1B**	mirecords; tarbase	Immunohistochemistry, In situ hybridization, **Luciferase reporter assay, Reporter assay**, Northern blot, **Western blot, qRT-PCR,** Microarray, PAR-CLIP, Degradome sequencing
22.	hsa-miR-222-3p	CEBPD	tarbase	Degradome sequencing
23.	hsa-miR-222-3p	COL5A2	mirtarbase	CLASH
24.	hsa-miR-222-3p	CYP1B1	tarbase	Degradome sequencing

25.	hsa-miR-222-3p	DAG1	mirtarbase	CLASH
26.	hsa-miR-222-3p	DBN1	mirtarbase	CLASH
27.	hsa-miR-222-3p	DDX20	tarbase	Degradome sequencing
28.	hsa-miR-222-3p	DERA	mirtarbase	CLASH
29.	**hsa-miR-222-3p**	**ETS1**	mirtarbase; tarbase	**Luciferase reporter assay,** Northern blot, **Western blot, qRT-PCR,** Microarray, Next Generation Sequencing (NGS), Degradome sequencing
30.	hsa-miR-222-3p	FOXC1	tarbase	Degradome sequencing
31.	hsa-miR-222-3p	FOXO1	mirtarbase	qRT-PCR//Western blot
32.	hsa-miR-222-3p	FRS2	tarbase	Degradome sequencing
33.	hsa-miR-222-3p	GAS5	mirtarbase	Immunofluorescence, Immunohistochemistry, **Luciferase reporter assay, Western blot, qRT-PCR**
34.	hsa-miR-222-3p	GJA1	mirtarbase; tarbase	Luciferase reporter assay, Immunofluorescence, Western blot, Degradome sequencing
35.	**hsa-miR-222-3p**	**MMP1**	mirecords; mirtarbase	Flow, **Luciferase reporter assay, Western blot, qRT-PCR,** Microarray
36.	hsa-miR-222-3p	NFATC3	tarbase	Degradome sequencing
37.	hsa-miR-222-3p	PCNT	mirtarbase	CLASH
38.	hsa-miR-222-3p	PDIK1L	mirtarbase	PAR-CLIP
39.	hsa-miR-222-3p	POMGNT1	mirtarbase	CLASH
40.	**hsa-miR-222-3p**	**PTEN**	mirecords; mirtarbase	FACS, Flow, Immunohistochemistry, **Luciferase reporter assay,** Northern blot, **Western blot, qRT-PCR,** Microarray
41.	hsa-miR-222-3p	RREB1	tarbase	Degradome sequencing
42.	hsa-miR-222-3p	SEC24C	mirtarbase	CLASH
43.	hsa-miR-222-3p	SELE	mirtarbase; tarbase	Luciferase reporter assay, Degradome sequencing
44.	hsa-miR-222-3p	SIX1	tarbase	Degradome sequencing
45.	hsa-miR-222-3p	SKIV2L	mirtarbase	CLASH
46.	**hsa-miR-222-3p**	**SOD2**	mirecords; mirtarbase	Flow, **Luciferase reporter assay, Western blot, qRT-PCR,** Microarray
47.	hsa-miR-222-3p	SPARC	tarbase	Degradome sequencing
48.	hsa-miR-222-3p	SQSTM1	tarbase	Degradome sequencing
49.	hsa-miR-222-3p	THBS1	tarbase	Degradome sequencing

50.	**hsa-miR-222-3p**	**TIMP3**	mirecords; tarbase	ELISA, Flow, Immunohistochemistry, In situ hybridization, **Luciferase reporter assay, Western blot, qRT-PCR**, Degradome sequencing
51.	hsa-miR-222-3p	TP53	mirtarbase	CLASH, Western blot
52.	hsa-miR-222-3p	TRIM44	mirtarbase	CLASH
53.	hsa-miR-222-3p	TUB	tarbase	Degradome sequencing
54.	hsa-miR-222-3p	TXN	mirtarbase	CLASH
55.	hsa-miR-222-3p	USP9X	mirtarbase; tarbase	CLASH, Degradome sequencing
56.	hsa-miR-222-3p	VCAN	tarbase	Degradome sequencing
57.	hsa-miR-222-3p	ZSWIM6	tarbase	Degradome sequencing

CLASH – Cross-linking, Ligation, and Sequencing of Hybrids, HITS-CLIP – High-Throughput Sequencing of RNA isolated by Cross-Linking Immunoprecipitation, PAR-CLIP – Photoactivatable Ribonucleoside-Enhanced Crosslinking and Immunoprecipitation, qRT-PCR – Quantitative Reverse-Transcriptase Polymerase Chain Reaction.

Supplementary Table S4. Top 10% predicted miRNA:gene pairs obtained in *silico* among the two upregulated miRNAs in both the aqueous humor and lens capsule of patients with glaucoma and 770 genes associated with glaucoma.

No.	miRNA miRBase ID	Gene symbol	Database	Database-specific probability value
1.	hsa-miR-193a-5p	*ABCC1*	microcosm	18.761
2.	hsa-miR-193a-5p	*ACVR1*	diana_microt	0.998
	hsa-miR-193a-5p	*ACVR1*	miranda	-1.335
	hsa-miR-193a-5p	*ACVR1*	mirdb	91.60862
	hsa-miR-193a-5p	*ACVR1*	pita	-15.73
	hsa-miR-193a-5p	*ACVR1*	targetscan	-0.487
3.	hsa-miR-193a-5p	*CEP57*	mirdb	91.20358
	hsa-miR-193a-5p	*CEP57*	targetscan	-0.484
4.	hsa-miR-193a-5p	*CNTNAP2*	pita	-10.4
5.	hsa-miR-193a-5p	*COL1A1*	pita	-14.15
	hsa-miR-193a-5p	*COL2A1*	pita	-10.58
	hsa-miR-193a-5p	*COL2A1*	pita	-14.55
6.	hsa-miR-193a-5p	*CYGB*	pita	-13.3
7.	hsa-miR-193a-5p	*DCLK1*	diana_microt	0.935
	hsa-miR-193a-5p	*DCLK1*	pita	-23.95
8.	hsa-miR-193a-5p	*DHDDS*	pita	-14
9.	hsa-miR-193a-5p	*FOXP3*	microcosm	17.8013
10.	hsa-miR-193a-5p	*GBA2*	diana_microt	0.82
	hsa-miR-193a-5p	*GBA2*	miranda	-1.2738
	hsa-miR-193a-5p	*GBA2*	miranda	-1.2827
	hsa-miR-193a-5p	*GBA2*	miranda	-1.2975
	hsa-miR-193a-5p	*GBA2*	targetscan	-0.675
11.	hsa-miR-193a-5p	*GNAQ*	diana_microt	0.907
12.	hsa-miR-193a-5p	*GNAS*	pita	-14.95
13.	hsa-miR-193a-5p	*GORASP1*	diana_microt	0.881
14.	hsa-miR-193a-5p	*GPATCH3*	microcosm	18.0022
15.	hsa-miR-193a-5p	*HSP90B1*	pita	-13.91
16.	hsa-miR-193a-5p	*KMT2A*	diana_microt	0.822
	hsa-miR-193a-5p	*KMT2A*	pita	-11.72

17.	hsa-miR-193a-5p	*LMX1B*	microcosm	17.6979
18.	hsa-miR-193a-5p	*MAPK10*	pita	-15.57
19.	hsa-miR-193a-5p	*MTOR*	pita	-13.2
20.	hsa-miR-193a-5p	*NDP*	pita	-11.51
21.	hsa-miR-193a-5p	*NTRK2*	diana_microt	0.814
22.	hsa-miR-193a-5p	*POMGNT1*	pita	-14.62
23.	hsa-miR-193a-5p	*PRPF4*	pita	-17.67
24.	hsa-miR-193a-5p	*RBFOX1*	pita	-12.62
25.	hsa-miR-193a-5p	*RUNX1T1*	pita	-15.18
26.	hsa-miR-193a-5p	*SBF2*	diana_microt	0.827
27.	hsa-miR-193a-5p	*SIGMAR1*	pita	-10.61
28.	hsa-miR-193a-5p	*TRPC5*	diana_microt	0.816
29.	hsa-miR-193a-5p	*VAV3*	pita	-10.57
30.	hsa-miR-193a-5p	*WNK1*	pita	-13.76
31.	hsa-miR-193a-5p	*XPR1*	pita	-18.92
32.	hsa-miR-222-3p	*AGBL5*	pita	-16.58
33.	hsa-miR-222-3p	*ANXA3*	miranda	-1.2404
34.	hsa-miR-222-3p	*ARHGEF7*	elmmo	0.658
35.	hsa-miR-222-3p	*ATF2*	pita	-11.45
36.	hsa-miR-222-3p	*BCHE*	miranda	-1.2247
37.	hsa-miR-222-3p	*BCOR*	microcosm	18.0191
	hsa-miR-222-3p	*BCOR*	pita	-11.87
38.	hsa-miR-222-3p	*CDKN1B*	diana_microt	0.995
	hsa-miR-222-3p	*CDKN1B*	elmmo	0.802
	hsa-miR-222-3p	*CDKN1B*	elmmo	0.735
	hsa-miR-222-3p	*CDKN1B*	elmmo	0.735
	hsa-miR-222-3p	*CDKN1B*	miranda	-1.2479
	hsa-miR-222-3p	*CDKN1B*	mirdb	99.27968321
	hsa-miR-222-3p	*CDKN1B*	targetscan	-0.515
	hsa-miR-222-3p	*CDKN1B*	targetscan	-0.534
39.	hsa-miR-222-3p	*CNR1*	diana_microt	0.825

40.	hsa-miR-222-3p	*CUX1*	elmmo	0.802
41.	hsa-miR-222-3p	*DBN1*	elmmo	0.58
42.	hsa-miR-222-3p	*DCLK1*	elmmo	0.495
43.	hsa-miR-222-3p	*DPYSL5*	elmmo	0.588
44.	hsa-miR-222-3p	*ETS1*	diana_microt	0.981
	hsa-miR-222-3p	*ETS1*	elmmo	0.802
	hsa-miR-222-3p	*ETS1*	elmmo	0.802
	hsa-miR-222-3p	*ETS1*	pita	-14.38
45.	hsa-miR-222-3p	*FBN1*	elmmo	0.722
46.	hsa-miR-222-3p	*FRS2*	pita	-9.96
47.	hsa-miR-222-3p	*GABPA*	pita	-10.95
48.	hsa-miR-222-3p	*KMT2A*	elmmo	0.58
	hsa-miR-222-3p	*KMT2A*	miranda	-1.0705
49.	hsa-miR-222-3p	*MAPK10*	elmmo	0.615
50.	hsa-miR-222-3p	*NFATC3*	diana_microt	0.806
	hsa-miR-222-3p	*NFATC3*	elmmo	0.666
51.	hsa-miR-222-3p	*NIPBL*	elmmo	0.826
	hsa-miR-222-3p	*NIPBL*	miranda	-1.2397
52.	hsa-miR-222-3p	*NTF3*	diana_microt	0.855
	hsa-miR-222-3p	*NTF3*	elmmo	0.802
	hsa-miR-222-3p	*NTF3*	miranda	-1.324
	hsa-miR-222-3p	*NTF3*	targetscan	-0.686
53.	hsa-miR-222-3p	*PIK3R1*	elmmo	0.735
	hsa-miR-222-3p	*PIK3R1*	mirdb	89.71156146
54.	hsa-miR-222-3p	*PTEN*	diana_microt	0.812
	hsa-miR-222-3p	*PTEN*	elmmo	0.529
55.	hsa-miR-222-3p	*PTPN11*	pita	-9.86
56.	hsa-miR-222-3p	*RIMS1*	pita	-10.35
57.	hsa-miR-222-3p	*RREB1*	elmmo	0.781
58.	hsa-miR-222-3p	*RUNX1T1*	diana_microt	0.846
59.	hsa-miR-222-3p	*S100B*	pita	-11.56

60.	hsa-miR-222-3p	*SEC24C*	miranda	-1.0849
61.	hsa-miR-222-3p	*SETD5*	pita	-10.05
62.	hsa-miR-222-3p	*SLC4A4*	elmmo	0.632
	hsa-miR-222-3p	*SLC4A4*	pita	-12.91
63.	hsa-miR-222-3p	*SNCA*	diana_microt	0.808
64.	hsa-miR-222-3p	*SRD5A3*	diana_microt	0.815
65.	hsa-miR-222-3p	*THBS1*	pita	-15.54
66.	hsa-miR-222-3p	*THSD7A*	elmmo	0.58
67.	hsa-miR-222-3p	*TIMP3*	elmmo	0.615
68.	hsa-miR-222-3p	*WWTR1*	microcosm	17.832
69.	hsa-miR-222-3p	*YAP1*	elmmo	0.495

Supplementary Table S5. Summary table of ClueGO result that shows related term list of target genes of miR-193a-5p and miR-222-3p. "% Associated genes" means percentage of associated genes in total genes of each term.

Term	Term p-Value	% Associated Genes	Associated Genes Found
postsynaptic actin cytoskeleton organization	0.0004951	16.67	[*ACTB, DBN1*]
regulation of miRNA transcription	<0.0001	5.97	[*ETS1, NFATC3, TP53, WT1*]
positive regulation of leukocyte adhesion to vascular endothelial cell	0.0021987	8.00	[*ETS1, SELE*]
regulation of vascular associated smooth muscle cell differentiation	0.0008937	12.50	[*NFATC3, SOD2*]
regulation of pentose-phosphate shunt	0.0001589	28.57	[*MTOR, TP53*]
DNA damage response, signal transduction by p53 class mediator resulting in cell cycle arrest	0.0011354	11.11	[*CDKN1B, TP53*]
morphogenesis of an epithelial sheet	<0.0001	6.25	[*DAG1, MTOR, PTEN, RREB1*]
regulation of epithelial to mesenchymal transition	<0.0001	5.21	[*COL1A1, DAG1, FOXC1, MTOR, PTEN*]
positive regulation of epithelial to mesenchymal transition	0.0004112	5.77	[*COL1A1, FOXC1, MTOR*]
wound healing, spreading of epidermal cells	<0.0001	14.29	[*MTOR, PTEN, RREB1*]
regulation of wound healing, spreading of epidermal cells	<0.0001	37.50	[*MTOR, PTEN, RREB1*]
regulation of animal organ formation	<0.0001	9.38	[*FRS2, SIX1, WT1*]
regulation of vascular associated smooth muscle cell proliferation	<0.0001	7.94	[*CDKN1B, FRS2, GJA1, PTEN, SOD2*]
positive regulation of brown fat cell differentiation	0.0010111	11.76	[*RREB1, SIX1*]
negative regulation of vascular associated smooth muscle cell proliferation	<0.0001	11.54	[*CDKN1B, PTEN, SOD2*]
positive regulation of vascular associated smooth muscle cell proliferation	0.0047779	5.41	[*FRS2, GJA1*]
anterior/posterior axis specification, embryo	0.0020264	8.33	[*FRS2, WT1*]
negative regulation of focal adhesion assembly	0.0014050	10.00	[*PTEN, THBS1*]
negative regulation of B cell proliferation	0.0012667	10.53	[*CASP3, PTEN*]
metanephric mesenchyme development	0.0010111	11.76	[*SIX1, WT1*]
negative regulation of cyclin-dependent protein serine/threonine kinase activity	<0.0001	9.68	[*CASP3, CDKN1B, PTEN*]
positive regulation of extrinsic apoptotic signaling pathway via death domain receptors	<0.0001	18.75	[*PTEN, THBS1, TIMP3*]
positive regulation of endothelial cell apoptotic process	0.0011354	11.11	[*AKR1C3, THBS1*]

Supplementary Table S6. MicroRNA profiles using intraocular samples from patients with glaucoma in previous studies and the present study.

Previous studies (Author)	Method	Sample	Disease (Glaucoma)	miRNAs	Reference
Present study	PCR array	Aqueous humor	OAG	**Up**: hsa-let-7a-5p, hsa-miR-125a-5p, hsa-miR-1285-3p, hsa-miR-181d-5p, hsa-miR-185-5p, hsa-miR-192-5p, hsa-miR-193a-5p, hsa-miR-194-5p, hsa-miR-195-5p, hsa-miR-210-3p, hsa-miR-221-3p, hsa-miR-222-3p, hsa-miR-25-3p, hsa-miR-31-5p, hsa-miR-365-3p, hsa-miR-512-5p, hsa-miR-542-3p, hsa-miR-9-5p, hsa-miR-92a-5p	
Present study	PCR array	Lens capsule	OAG	**Up**: hsa-let-7e-5p, hsa-miR-193a-5p, has-miR-222-3p	
Cho et al., 2022	RNA sequencing	Aqueous humor	XFG	**Up**: hsa-miR-30d-5p and hsa-miR-320a<br>**Down**: hsa-miR-3156-5p, hsa-miR-4458, hsa-miR-6717-5p, hsa-miR-6728-5p, hsa-miR-6834-5p, hsa-miR-6864-5p, hsa-miR-6879-5p, hsa-miR-877-3p, hsa-miR-548e-3p, **hsa-miR-6777-5p**	33
Cho et al., 2022	RNA sequencing	Aqueous humor	NTG	**Up**: hsa-let-7a-5p, hsa-let-7c-5p, hsa-let-7f.-5p, hsa-miR-192-5p, hsa-miR-10a-5p, hsa-miR-10b-5p, hsa-miR-375, hsa-miR-4510, hsa-let-7b-3p, hsa-miR-222-3p<br>**Down**: hsa-miR-4639-5p, **hsa-miR-6777-5p**	33
Seong et al., 2021	RNA sequencing	Aqueous humor	NTG	**Up**: hsa-let-7a-5p, hsa-let-7c-5p, hsa-let-7f-5p, hsa-miR-192-5p, hsa-miR-10a-5p, hsa-miR-10b-5p, hsa-miR-375, **hsa-miR-143-3p**	38
Hubens et al., 2021	Small RNA sequencing	Aqueous humor	POAG	**Up**: hsa-miR-30a-3p, **hsa-miR-143-3p**, hsa-miR-211-5p, hsa-miR221-3p<br>**Down**: hsa-miR-92a-3p, hsa-miR-451a, hsa-miR-486-5p	32
Kosior-Jarecka et al., 2021	RT-PCR/PCR array	Aqueous humor	POAG	No significantly differential expression<br>(Tended to be downregulated: miR-6515-3p)	20
Kosior-Jarecka et al., 2021	RT-PCR/PCR array	Aqueous humor	XFG	No significantly differential expression<br>(Tended to be downregulated: miR-1260b)	20
Kosior-Jarecka et al., 2021	RT-PCR/PCR array	Aqueous humor	PACG	No significantly differential expression<br>(Tended to be downregulated: miR-1260b)	20
Hindle et al., 2019	PCR array	Aqueous humor	POAG	**Up**: hsa-miR-637, hsa-miR-99b-3p, hsa-miR-4725-3p, hsa-miR-4724-5p, hsa-miR-4538, hsa-miR-433-3p	13
Hindle et al., 2019	PCR array	Aqueous humor	XFS+XFG	**Up**: hsa-miR-637, hsa-miR-99b-3p, hsa-miR-4725-3p, hsa-miR-4724-5p, hsa-miR-4538, hsa-miR-433-3p	13
Liu et al., 2018	NGS	Aqueous humor	POAG	**Up**: 73 miRNAs<br>**Down**: 15 miRNAs<br>(Upregulated in the severe group: hsa-miR-205-5p, hsa-miR-206,	34

				hsa-miR-16-5p, hsa-miR-501-3p, hsa-miR-409-3p, hsa-miR-200a-3p, hsa-miR-200b-3p, hsa-miR-382-5p, hsa-miR-543, hsa-miR-136-3p, hsa-miR-30c-2-3p, hsa-miR-139-5p, hsa-miR-340-5p, hsa-miR-488-3p, hsa-miR-202-5p, hsa-miR-369-5p)	
Drewry et al., 2018	NanoString	Aqueous humor	POAG	**Up**: hsa-miR-451a, hsa-miR-302d-3p<br>**Down**: hsa-miR-125b-5p	35
Drewry et al., 2018	NanoString	Aqueous humor	XFG	**Up**: hsa-miR-122-5p, hsa-miR-320e, hsa-miR-3144-3p, hsa-miR-630<br>**Down**: hsa-miR-320a	35
Jayaram et al., 2017	PCR array<br>*RT-PCR (low density PCR array)*	Aqueous humor	POAG	**Up**: hsa-miR-518d, hsa-miR-143<br>**Down**: hsa-miR-660	36
Tanaka et al., 2014	Microarray	Aqueous humor	POAG+PACG+XFG	**Up**: hsa-miR-4484, hsa-miR-6515-3p, hsa-miR-3663-3p, hsa-miR-4433-3p, hsa-miR-6717-5p, hsa-miR-4725-3p, hsa-miR-1202, hsa-miR-3197<br>**Down**: hsa-miR-4507, hsa-miR-3620-5p, hsa-miR-5001-5p, hsa-miR-6132, hsa-miR-4467, hsa-miR-187-5p, hsa-miR-6722-3p, hsa-miR-4749-5p, hsa-miR-1260b, hsa-miR-4634	37

Up, upregulated; Down, downregulated; hsa, Homo sapiens species; miR, microRNA; NTG: normal-tension glaucoma; OAG, open-angle glaucoma; PACG: primary angle closure glaucoma; PCR: polymerase chain reaction; POAG, primary open-angle glaucoma; XFG: exfoliation glaucoma; XFS: exfoliation syndrome
